# Screen Printed Reflective Electrochromic Displays for Paper and Other Opaque Substrates

**DOI:** 10.1021/acsaom.2c00140

**Published:** 2023-01-26

**Authors:** Kathrin Freitag, Robert Brooke, Marie Nilsson, Jessica Åhlin, Valerio Beni, Peter Andersson Ersman

**Affiliations:** † RISE Research Institutes of Sweden, Digital Systems, Smart Hardware, Printed, Bio- and Organic Electronics, Bredgatan 33, SE-60221 Norrköping, Sweden

**Keywords:** electrochromic displays, printed electronics, paper electronics, organic electronics, PEDOT:PSS

## Abstract

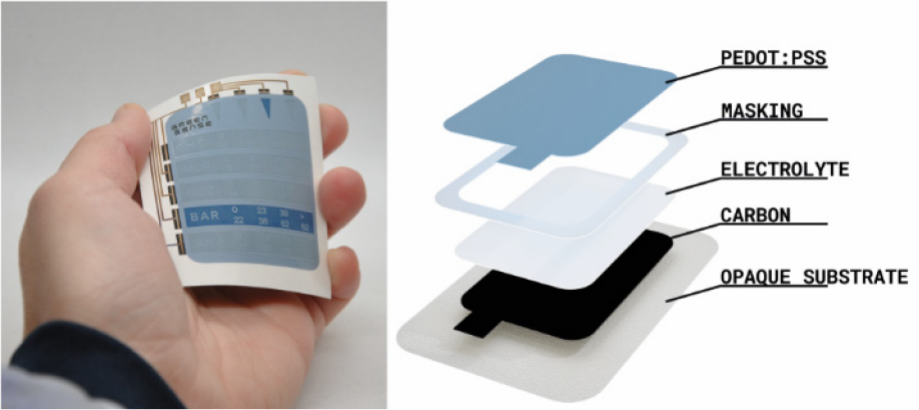

Paper electronics is a viable alternative to traditional electronics, leading to more sustainable electronics. Many challenges still require solutions before paper electronics become mainstream. Here, we present a solution to enable the manufacturing of reflective all-printed organic electrochromic displays (OECDs) on paper substrates; devices that are usually printed on transparent substrates, for example, plastics. In order to operate on opaque paper substrates, an architecture for reversely printed OECDs (rOECDs) is developed. In this architecture, the electrochromic layer is printed as the last functional layer and can therefore be viewed from the print side. Square shaped 1 cm^2^ rOECDs are successfully screen printed on paper, with a high manufacturing yield exceeding 99%, switching times <3 s and high color contrast (ΔE* > 27). Approximately 60% of the color is retained after 15 min in open-circuit mode. Compared to the conventional screen printed OECD architectures, the rOECDs recover approximately three times faster from storage in a dry environment, which is particularly important in systems where storage in low humidity atmosphere is required, for example, in many biosensing applications. Finally, a more complex rOECD with 9 individually addressable segments is successfully screen printed and demonstrated.

## 1 Introduction

Sustainability and environmental concerns are now on the forefront of many research activities. The field of electronics is one of the largest contributors to unsustainable waste with projections showing a troubling future.^1^ Paper electronics has the potential to provide more sustainable electronics by having lower CO_2_ footprint, facilitating the recycling/recovery of materials and avoiding the landfill end of life.^2,3^ The field of paper electronics is advantageous for any printed electronics application, if only due to the potential reduction in cost compared to standard PET substrates (∼0.1 cent dm^–2^ vs 2 cent dm^–2^) and the ability for circular economy.^3^ In addition to these advantages, combining paper substrates with printing technologies can allow large-area manufacturing while at the same time keeping costs low by high throughput manufacturing,^4,5^ and it is also possible to pattern graphene-based electrical conductors on paper substrates via laser irradiation.^6^ One area of printed electronics that has reached a high level of matureness is printed displays.^7,8^ As a result, printed smart electronic labels have received growing interest due to the drive of new, inexpensive Internet of Things (IoT) devices and the need for low-cost informative communication.^9^ Additionally, displays and indicators accompanying cheap sensors have permitted the development of many new platforms for testing of drugs, diseases or environmental conditions.^10,11^

While many display technologies exist, each possesses certain advantages and disadvantages.^12,13^ These include more conventional liquid crystal and light emitting diode displays^13,14^ to simpler and cheaper thermochromic and electrochromic displays.^15,16^ Due to their simplicity in design and ability to be printed, thermochromic and electrochromic displays are most suited to accompany cheap, printed sensor technologies. Electrochromic displays have certain advantages over other display technologies, including low voltage operation, relatively long retention time (optical memory), large viewing angle, and their ability to be manufactured by printing technology.^17^ Many reports have shown impressive electrochromic properties within the scientific literature,^18−20^ even on flexible substrates,^21,22^ however, only the use of the conductive polymer poly(3,4-ethylenedioxythiophene):polystyrenesulfonate (PEDOT:PSS) has been commercialized.^7,8^

PEDOT has been extensively studied in the scientific literature, partially due to its excellent electrical and electrochromic properties.^19,23^ The combination with PSS has allowed the PEDOT to become processable from water-based ink formulations and permitted deposition via various printing technologies, such as inkjet printing,^24^ slot die coating,^25^ and screen printing.^26^ Organic electrochromic displays (OECDs) incorporating PEDOT:PSS have been fabricated using all the above printing technologies,^27,28^ however, only screen printing has been utilized in the manufacturing of commercially available OECDs.^7,8^ Screen printing, through the abilities of excellent alignment and resolution (∼100 μm), allows printing of several functional layers for all-printed OECDs and can be adapted for both sheet-by-sheet printing and roll-to-roll pilot production.^29^

Previously reported all-printed OECDs with conventional architecture, in which the electrochromic electrode material is deposited onto the transparent substrate, show excellent performance,^30^ however, they cannot be employed in combination with opaque substrates, for example, paper, since the color change in such devices is observed through the transparent substrate. Therefore, two options are available for transitioning this display technology to the field of paper electronics: lateral displays or displays with reverse print order; the former is a coplanar device architecture, while in the latter the electrochromic electrode material is instead deposited on top of the electrolyte. Unfortunately, lateral displays suffer from slow switching response. This is due to the relatively low ionic conductivity in printed and solidified electrolytes, which typically give rise to a gradual, or curtain, switching behavior of the display segments. Additionally, the active display area is decreased in lateral displays, due to the area required by the counter electrode.^31^ For these reasons, a reverse OECD architecture (rOECD) is more suitable.

Although similar designs of rOECDs have been reported in the scientific literature,^21,28,32^ to the best of our knowledge, none have shown the fabrication of rOECDs using only printing technologies. Lamination of two sheets is usually described within the scientific literature, mostly due to difficulties in overprinting on top of a previously deposited electrolyte layer. An overprintable electrolyte implies certain requirements. Besides providing ions with sufficient mobility for the electrochromic switch, the electrolyte should also withstand the mechanical forces applied by the screen printing tools during the printing process. In addition to this, the cured electrolyte layer needs to be chemically resistant to the subsequently screen printed ink formulation, that is, it must not be dissolved by the functional ink formulation deposited on top of the electrolyte. Within this report, we utilize a curable electrolyte, which is overprintable with PEDOT:PSS used as the electrochromic electrode material, to successfully demonstrate the concept of all-printed rOECDs.

Another gap in the OECD technology is the fabrication on paper substrates, and especially fabrication using solely printing technologies. Previous examples of electrochromism on paper substrates have either been incorporated as lateral displays or laminated into active matrix addressed displays.^33−35^ Other examples have used the paper itself within the electrochromic device as a separator.^36,37^

From a manufacturing point of view, an impressive work was recently reported by Hakola et al., who presented a roll-to-roll screen printed paper electronic platform that incorporated communication via a printed antenna and an OECD.^38^ While the schematic of the device was not technically a reverse display, the success of this prototype shows that paper electronic platforms can be manufactured in a cost-effective roll-to-roll high volume fashion, allowing for low-cost and sustainable one-time use paper electronic devices. That being said, the OECD presented in the manuscript is very simple in design, only a circle that shows a light blue or dark blue color depending on the redox state. By patterning the electrochromic display into segments or pixels, more information can be extracted from the devices, such as values from sensor components, battery life, or complex imagery.^30,39^

**Figure 1:**
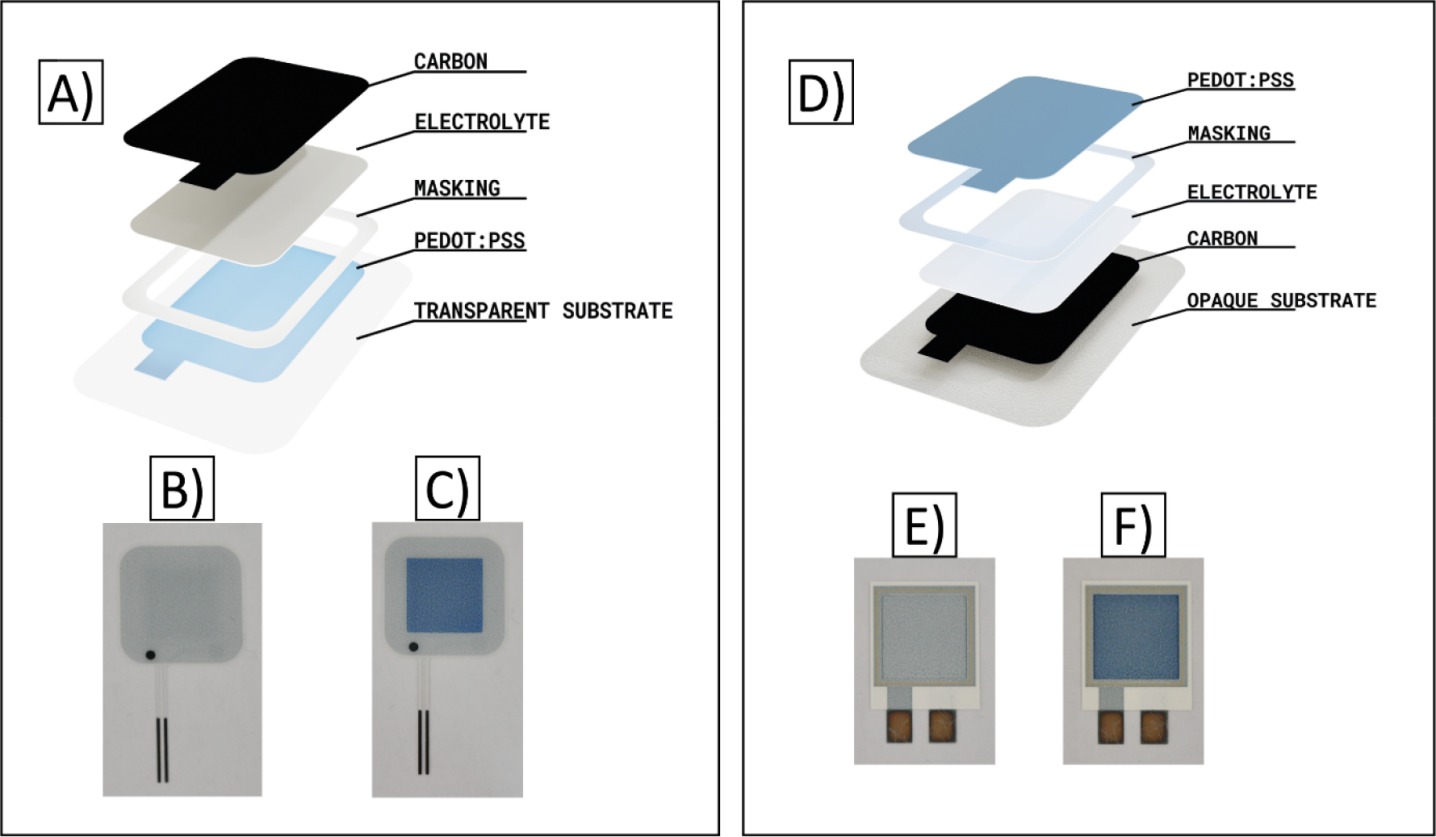
Schematics showing the difference between conventional and reverse OECD architectures. A) The conventional OECD architecture that can only be printed on transparent substrates vs D) the reverse OECD architecture developed and reported herein, allowing for screen printing on opaque substrates. The photographs in B) and C) show the OFF and ON states of a conventional OECD architecture, while E) and F) show the OFF and ON states of a reverse OECD. The switchable segment area in B), C), E), and F) is 10 × 10 mm^2^.

Within this report we present all-printed, patterned rOECDs, including subsequent screen printing of the electrochromic material on top of the electrolyte layer,^40^ allowing the use of opaque paper substrates and thereby bringing the commercialized technology of conventional OECDs into the field of paper electronics. The difference between conventional and reverse OECDs is elucidated in the diagrams of Figure 1. In this report we also show that while the conventional OECDs on transparent (plastic) substrates have slightly better color contrast values, the recovery time from low humidity environments is improved with the reverse OECD architecture. Importantly, we highlight that this reverse display architecture shows performances that are independent of the substrate (for example, paper or plastic) on which they are printed.

Finally, to highlight the potentiality of the proposed technology, a complex rOECD with 9 segments was developed to be implemented, in the long term, within a paper-based Point of Care (PoC) biosensor platform. This rOECD also contains a new method of showing a patterned display by the incorporation of a graphical layer and a color filter layer in order to modify the perceived color of the entire display.

## 2 Experimental Section

### 2.1 Screen Printing

Plastic substrates (*Hostaphan*, 125 μm thick PET, purchased from *Mitsubishi*) and paper board (KKC paperboard grammage 274 g m^–2^, 400 μm thickness, *Klabin*) were used as substrates for the manufacturing of the rOECDs. The surface energy of the *Klabin* paperboard was 28.5 ± 2.3 mN m^−1^ (disperse 26.7 ± 1.2 mN m^−1^, polar 1.8 ± 1 mN m^−1^). Successful cross hatch tests were also performed to evaluate the adhesion of screen printed silver lines, that is, no silver was removed from the *Klabin* paperboard. PET films were preshrunk in a belt oven for 6 min at 130 °C to improve the heat stability of the substrates. Similarly, the paper boards were preheated at 120 °C for 4 min prior to screen printing. Due to the tendency of the paper to change dimensions upon humidity uptake (up to 0.5%, see Figure S1 in the Supporting Information), the paper substrates were additionally run through the oven for 2 min at 120 °C directly before every printing step. Furthermore, to minimize the buckling of the paper substrate and facilitate the printing process, the paper boards were hot pressed at 130 °C for approximately 40 s.

Screen printing of the different layers was performed using a DEK Horizon 03iX screen printer and frames with polyester meshes. Screens with different mesh counts (threads per centimeter and thread diameter) were used in the different layers: 100–40 for the electrolyte, 120–34 for PEDOT:PSS, carbon and silver, and 140–31 for the insulating layers. The screen layout is shown in Figure S2. The approximate thicknesses of the screen printed layers are carbon 9 μm, electrolyte 13 μm, insulator 15 μm, PEDOT:PSS 0.5 μm and silver 11 μm.^41^

A schematic of the rOECD architecture is presented and compared with the conventional OECD architecture in Figure 1. For the reverse OECD architecture presented herein, the first layer screen printed onto the substrate was a carbon paste (7102 purchased from *DuPont*), which served as the counter electrode. Thereafter two layers of electrolyte (*E003*, a polyelectrolyte-based ink formulation for screen printing, commercially available from *RISE*) were screen printed, including subsequent curing after each screen printing step. The reason for printing two layers is to minimize the risk of pinholes. To define the active areas of the display segments, two layers of an insulator (*UVSF 173* purchased from *Marabu*) were deposited in the following screen printing steps, including a subsequent curing step after each screen printing step. As the pixel electrode, or color changing electrode, two layers of an ink containing PEDOT:PSS (poly(3,4-ethylenedioxythiophene) doped with poly(styrene sulfonic acid)), *S V4* (purchased from *Clevios*) or *EL-P 5015* (purchased from *Agfa*), were screen printed on top, and into the cavities, of the insulating layer. These are all the functional layers required to enable electrochromic switching in the display segments, but to lower the overall resistance of the display, and therefore to shorten the switching time, a silver conductor (*Ag 5000* from *DuPont*) was subsequently screen printed along the outline of the display segments. Two layers of an insulating ink were then screen printed on top, one opaque layer for the graphical pattern (color matched *UVSW*-based ink provided by *Marabu*) and one transparent layer for the mechanical protection of the display (*UVSW 904* purchased from *Marabu*). The green-colored *UVSW*-based ink used as mechanical protection in some of the rOECDs (Figure 7) was color matched and provided by *Marabu*. The different inks were cured prior to the printing of the following layer; the insulating layers were cured with UV light, at a dose of approximately 800 mJ cm^–2^, while the other layers were heat cured at 120 °C for 2 min. The electrolyte layers were heat treated at 60 °C for 2 min and then cured with UV light (∼800 mJ cm^–2^).

Two different rOECD types were manufactured: Type A, containing one layer each of the S V4 and EL-P 5015 PEDOT:PSS inks as electrochromic layers, and Type B, containing two layers of the S V4 PEDOT:PSS ink.

### 2.2 Electrical Measurements

All measurements were performed, if not stated otherwise, at ambient room condition (20–23 °C and 45–55%RH). The current vs time characteristics of the displays were performed using a semiconductor parameter analyzer (*HP/Agilent 4155B*). Prior to recording a measurement, the displays were initialized by switching them (at least 3 times) between their reduced and oxidized states; this was achieved by alternately applying 3 V and −3 V for a few seconds. To bring the rOECD to its reduced state, a constant voltage of 3 V was then applied to the counter electrode for 10 s, while the current was recorded. A laser was used to irradiate the center of the rOECD segment, and the scattered light was recorded by a photodiode to monitor the optical changes in the display during the switch experiments. To reduce background noise in the measurement, a cardboard box was used to cover the measurement setup, leaving only the laser light on the rOECD.^42^

### 2.3 Color Contrast Measurements

A spectrophotometer (*Mercury*, *Datacolor*) was used to measure the color contrast in the CIE L*a*b* color space.^32^ The oxidized state of the display was used as a reference. To record this, the display was oxidized to its OFF state by applying −3 V to the counter electrode for a few seconds, until the display reached its saturated white state. The white color results from the white electrolyte behind the nearly transparent oxidized PEDOT:PSS. For the observation of the maximum color contrast and the retention time, the displays were switched to their saturated reduced blue colored ON state by applying 3 V to the counter electrode for ∼20 s, the display was then left in open-circuit mode while the color coordinates were regularly recorded over time. The maximum color contrast (ΔE*) was then obtained as the difference between the color coordinates obtained for the display in the ON state and those from the OFF state. The color retention time was instead determined by comparing the initial color contrast of the ON state with the remaining color contrast recorded at each subsequent measurement of the OECD.

### 2.4 Pouch Test

To simulate storage in dry environments (below 5%RH), both conventional OECDs and rOECDs were sealed in an aluminum pouch together with a desiccant (0.5 g silica gel, dried at 120 °C for 30 min prior usage). The displays were stored in the pouch for at least 5 days.

The experiments in dry environment were performed on displays of Type B. The color contrasts obtained for rOECDs were compared with those obtained for conventional OECDs manufactured onto transparent plastic substrates, see Figure 1. The same inks were used for both display architectures, only the printing order of the functional layers was changed.

## 3 Results and Discussion

rOECDs were screen printed as described above, with a geometry of 1 × 1 cm^2^; this was chosen to meet the dimension requirements of the spectrophotometric probe used for the evaluation of the color contrast. 130 rOECDs with an active area of 1 cm^2^ were printed with a manufacturing yield of 100%, while 200 rOECDs of a larger design, with 9 segments each (with a total active area of 16 cm^2^), were screen printed with a manufacturing yield of 97%. The values reported are based on the manufacturing yield of the complete display; hence, the segment manufacturing yield of this print batch is with 99.7% even higher. The manufacturing yield was determined via both visual inspection of the OECDs and from the analysis of the current levels in the current vs time characteristics (see Experimental Section), and all dismissed displays were due to short-circuits in the rOECD. Such short-circuits are most often originating from pinholes in the electrolyte, but poor step coverage and broken conductors may also cause erroneous displays. Reduction of PEDOT:PSS not only changes the optical absorption characteristics, it also lowers the electronic conductivity by several orders of magnitude. Therefore, once the fully reduced blue colored state is reached, very low current throughput is expected in the device. Any sign of elevated current levels toward the end of the current vs time measurement is therefore an indication of potential short-circuits in the rOECDs. Furthermore, the stability of the rOECDs was tested by switching them on and off multiple times. The initial performance was compared with respect to both the color contrast and the current vs time switching behavior after 100 switch cycles; no significant change in the appearance or switching behavior could be observed, see Table S1 and Figure S3.

In the end of this section (Results and Discussion), we show that no difference in the display behavior was observed, whether printed on paper or PET. Therefore, all experiments described in this manuscript were performed on rOECDs printed on a PET foil for ease of handling, unless otherwise stated. Printing on PET allows for a simpler printing procedure, as no extra preheating step is needed before each layer is printed, as described in Figure S1.

### 3.1 Reverse vs Conventional OECD Architectures

**Figure 2:**
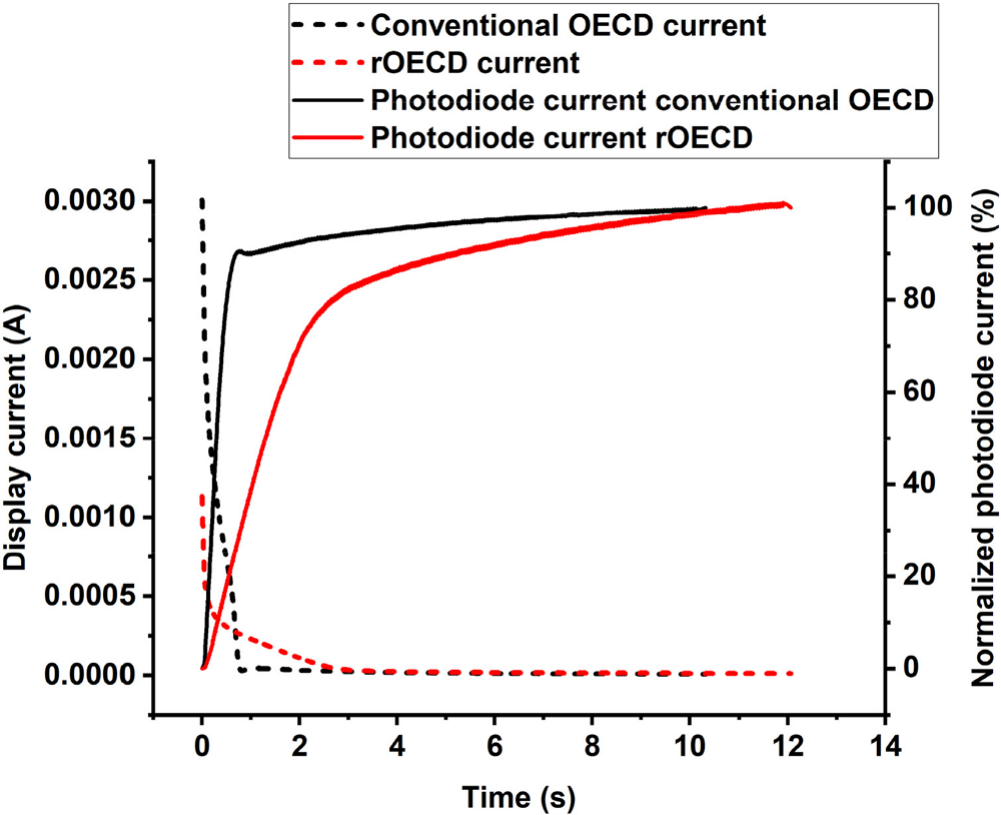
Switching behavior of the conventional and the reverse OECD architecture is shown in the black and red curves, respectively. The current vs time characteristics, with a constant voltage of 3 V applied to the counter electrode of the display (turning it into its blue colored ON state), are shown in dashed lines. The change in color is indicated by the photodiode current (solid lines) for the respective OECD architecture. The conventional and the reverse OECD architecture had a switching time of 0.75 and 2.8 s, respectively.

The performance of the novel rOECD architecture presented herein was compared to that of the conventional OECD.^43−45^ For this, OECDs of both reverse and conventional architectures with two layers of the S V4 PEDOT:PSS ink were used (referred to as Type B displays). Figure 2 shows the typical current vs time plots, recorded during the ON switch, for the conventional and reverse OECD and their change in color (recorded with a photodiode as described in the Experimental Section) during switching. The switching time is determined from the clear transition in the photodiode current. This also corresponds to a display current dropping down to a minimum, as the PEDOT:PSS is highly resistive in its reduced state, thereby indicating that the display is fully switched ON. The switching time was 0.75 s for the conventional OECD and 2.8 s for the rOECD. The most plausible explanation for the slower switch in the rOECD is due to increased resistance in the PEDOT:PSS layer when printed on top of the electrolyte, as compared to PEDOT:PSS printed onto the passive plastic substrate in the conventional OECD. The polar solvent of the PEDOT:PSS ink formulation most likely interacts with the electrolyte, through partial solvation, resulting in increased resistance that does not only lead to a longer switching time but also to lower current values in the beginning of the switch.

**Figure 3:**
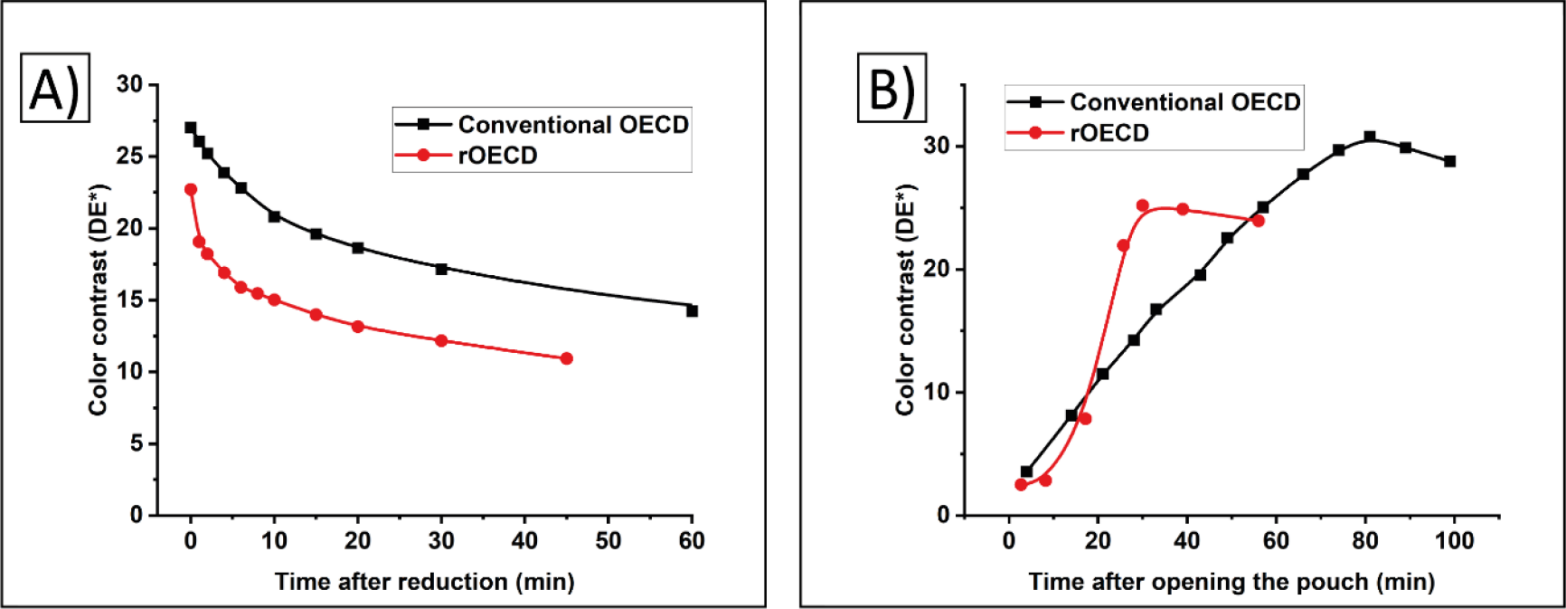
Retention time and the recovery time after storage in dry environment investigated by comparing reverse and conventional OECD architectures. A) The maximum color contrast (ΔE*) of the display is obtained at *t* = 0 min, when the display is reduced with 3 V applied to the counter electrode. The subsequent drop in color contrast when the display is left in open-circuit mode is shown with respect to time after switching the display to its saturated reduced state. The initial ΔE* is 23 for the rOECD and 27 for the conventional OECD. B) Recovery of the OECDs after storage in a dry pouch (<5%RH). The respective graph shows the maximum reachable color contrast upon reducing with 3 V for 20 s at different times after removing the displays from the dry environment inside a pouch including a desiccant.

The maximum color contrast and the retention time were evaluated by switching both a conventional OECD and a rOECD to their maximum reduced blue colored state and then measuring the color contrast over time, with the displays in open-circuit mode. The results of these evaluations are shown in Figure 3A. The conventional OECD reached a higher color contrast with a ΔE* of 27 compared to the rOECD with a ΔE* of 23. The color retention of the rOECDs was relatively poor, since most of the color is fading already during the first minutes, with 66% of the color contrast remaining after 10 min. The conventional OECD, however, retained the color contrast for a longer time, with 77% remaining after 10 min.

In recent years, the integration of OECDs in a variety of printed electronic applications is attracting more and more interest. One of the most attractive, and at the same time challenging, field of application for OECDs is those of single use PoC devices.^46^ Biomolecules used in PoC devices are often sensitive to humidity. To improve their shelf life, these devices are therefore often sealed in an airtight pouch together with a desiccant where an environment below 5%RH is created. While this is beneficial for the biomolecules, the extremely dry environment significantly affects the performance of the OECDs by drying out the electrolyte and the electrochromic layers. In dry OECDs the ionic mobility, instrumental for the electrochemical switching of the electrochromic material, is suppressed. Therefore, to become appealing for such applications, it is important that the OECDs are either encapsulated tightly (no loss of moisture) or that they can absorb humidity as fast as possible once the dry pouch has been opened, to regain its electrochromic functionality within a reasonable time span.

To test the benefit of the rOECD architecture for this application, OECDs of both architectures (reverse and conventional) were stored in a sealed metallic pouch with a desiccant. Both OECD architectures were printed with two layers of S V4 according to the Type B material combination. Pouches were opened at the earliest 5 days after sealing. The maximum reachable color contrast of the display was then measured, following the protocol described previously, at different times after opening the pouch. The room condition in which the measurements were recorded was approximately 20 °C and 50%RH. The resulting color contrast values with respect to time after opening the pouch are shown in Figure 3B.

The display printed according to the conventional architecture required ∼80 min to reach full color contrast, while the display printed according to the reverse architecture only required ∼30 min. Hence, the electrolyte and the electrochromic layer seem to absorb water much faster in the rOECD architecture presented herein. The most plausible explanation for this behavior is that the electrolyte and the PEDOT:PSS layers are “encapsulated” by the plastic substrate and the counter electrode in the conventional OECD, while they instead are printed as the final functional layers in the rOECD architecture, that is, they are more directly exposed to the ambient environmental condition.

### 3.2 Type A vs Type B Reverse OECD Architectures

**Figure 4:**
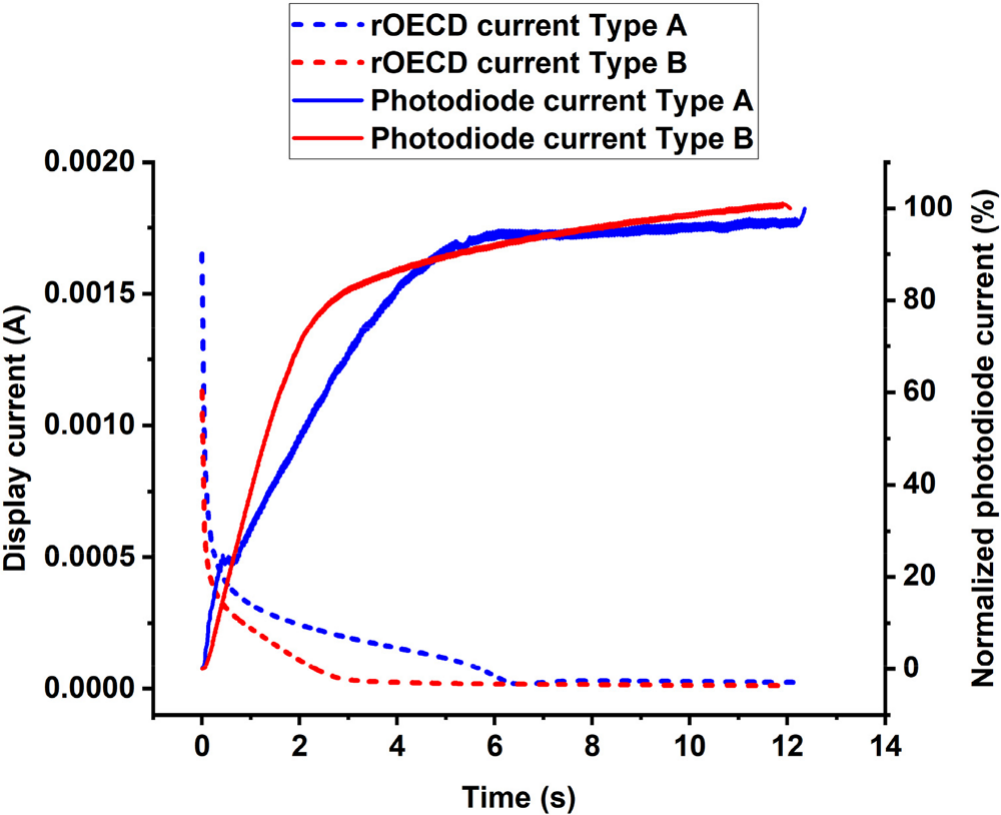
Switching behavior of the different types of rOECDs. The current vs time characteristics (dashed lines) of the Type A (blue) and Type B (red) rOECDs when a constant voltage of 3 V is applied to the counter electrodes of the OECDs, thereby switching them to their reduced blue colored state (ON). The change in color is indicated with the photodiode current (solid lines) for the respective OECD. The switching time was approximately 5.0 and 2.8 s for Type A and Type B, respectively.

With the aim of improving the color contrast in the rOECDs, different combinations of PEDOT:PSS inks (S V4 and EL-P 5015; see Experimental Section), were explored. A combination of one layer of EL-P 5015 on top of a S V4 layer (herein referred to as Type A display) was printed to obtain a higher color contrast value compared with the displays printed with two layers of S V4 (referred to as Type B display). Printing two layers of EL-P 5015 would lead to a too dark display in the oxidized OFF state, therefore this option was not further evaluated. Figure 4 shows typical current vs time plots recorded during the ON switching of Type A and Type B rOECDs. The switching time observed with the photodiode was ∼5 s for display Type A and <3 s for display Type B.

**Figure 5:**
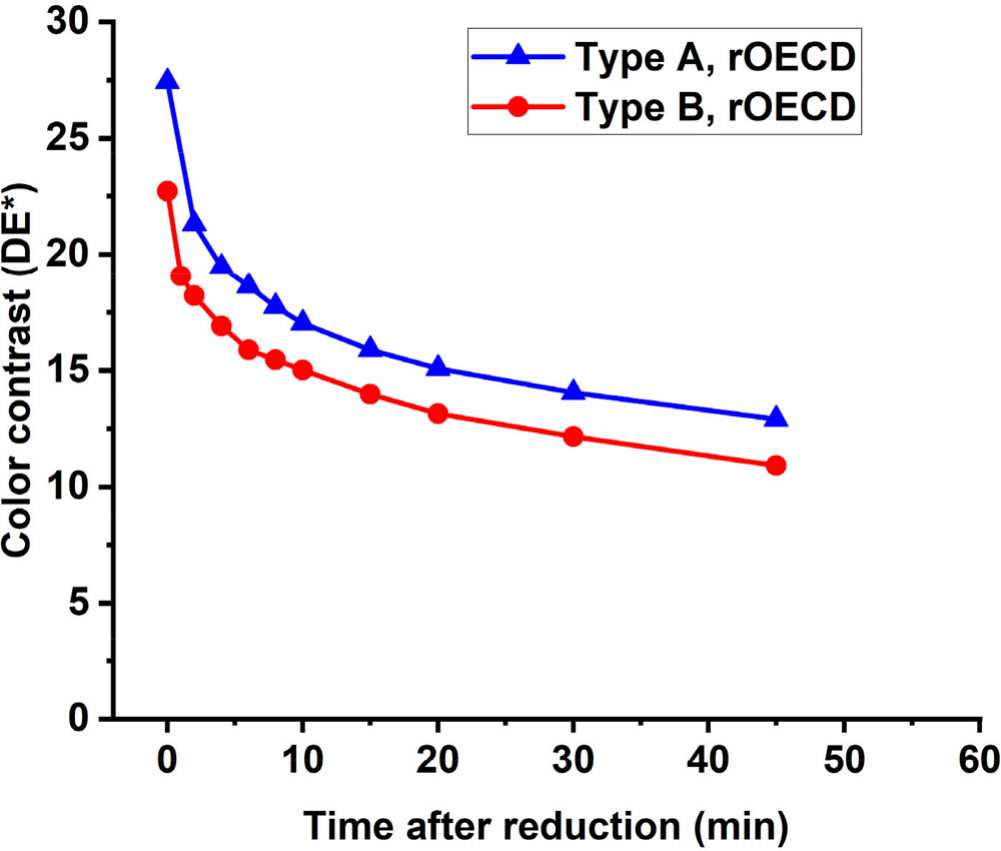
Maximum color contrast and color retention of the Type A and Type B rOECDs. The maximum color contrast (ΔE*) of the display is obtained at *t* = 0 min, when the display is reduced with 3 V. The subsequent drop in color contrast when the display is left in open-circuit mode is shown with respect to time after switching the display to its saturated reduced state. The maximum color contrast ΔE* was 27 and 23 for the rOECD Type A and Type B, respectively.

The maximum color contrast and the retention time were also evaluated for rOECDs of both Type A and B. By switching the rOECDs to their maximum reduced blue colored state by applying 3 V for 20 s, a color contrast ΔE* of 27 was recorded for Type A, while a ΔE* of 23 was obtained for Type B. This difference in color contrast might be due to a higher solid content of the EL-P 5015 ink compared to S V4, which in turn leads to a larger amount of PEDOT:PSS in the electrochromic film in Type A displays, resulting in the increased color contrast at the cost of switching time. After switching the OECDs to their blue colored state, they were kept in open-circuit mode and the remaining color contrast was measured with respect to time; the typical results obtained for Type A and Type B OECDs are shown in Figure 5.

The color retention of both rOECD types was relatively poor, since most of the color is fading already during the first minutes, but with 62 and 66% of the initial ΔE* still remaining after 10 min for Type A and Type B, respectively. Thereafter, the remaining color contrast becomes more stable with respect to time. Generally, ΔE* values exceeding 10 are considered easily detectable by the human eye; this makes the developed rOECDs adequate for displaying information for at least ∼45 min without the need of a refresh pulse.

### 3.3 Paper vs PET Substrate

**Figure 6:**
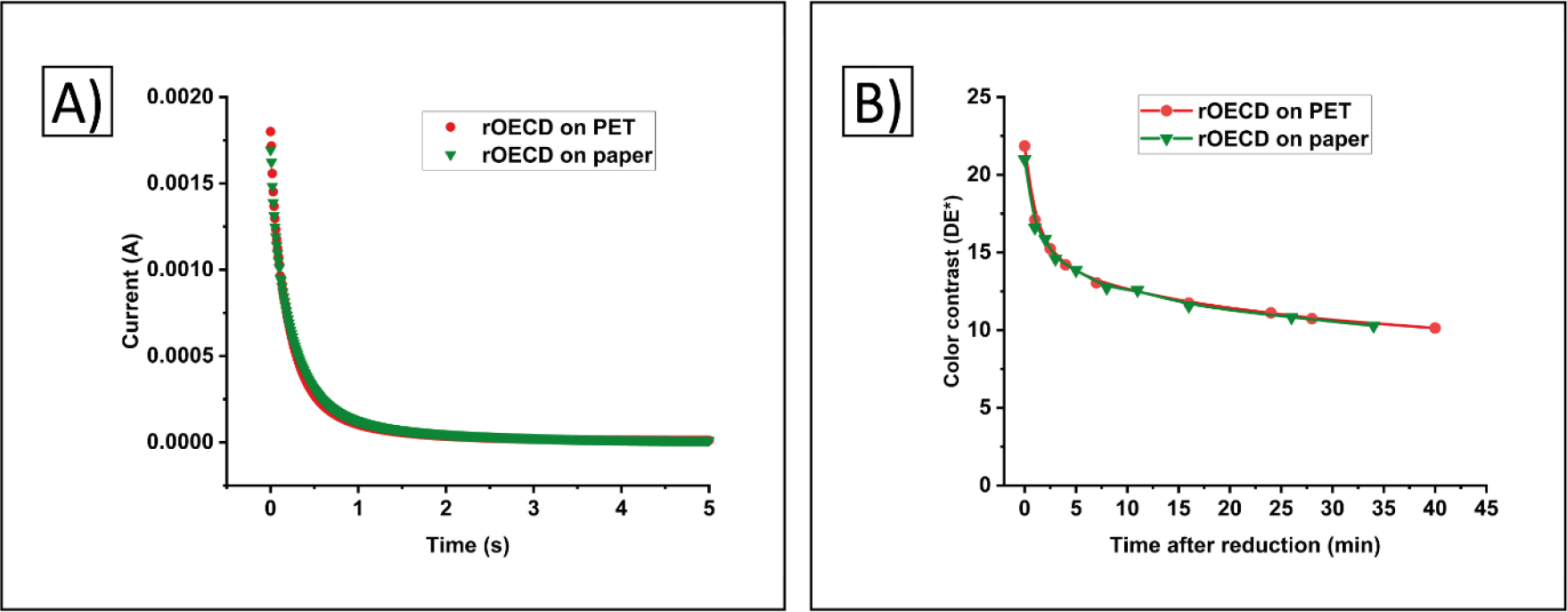
Comparison of reverse OECDs printed on PET and paper substrates. A) The current vs time characteristics when applying 3 V to reduce the displays to their ON states. B) The maximum color contrast of the rOECDs upon reduction is given at *t* = 0 (ON state), and the color retention when keeping the rOECDs in open-circuit mode is provided by the color contrast vs time.

Screen printing on paper substrates brings additional challenges to the manufacturing process. More specifically, paper substrates are much more prone to water uptake, in comparison with plastic substrates, and therefore suffer more from dimensional changes (Figure S1). This makes the alignment of multilayered printed electronic devices more difficult, especially on large-area substrates, thereby resulting in lower manufacturing yield. Such expansion of the paper substrate can be mitigated by thermal treatment prior to every screen printing step, and possibly also hot pressing to avoid buckling of the substrate. But to minimize the number of processing steps in the development of the rOECD architecture, most of the rOECDs were screen printed on plastic substrates, and all the above-mentioned experiments were performed on rOECDs on PET substrates. However, rOECDs screen printed on paper substrates were also produced in the same printing batch. Figure 6 shows that no substantial difference in the switching behavior of the rOECDs printed on the different substrates could be observed, neither in the current vs time characteristics (Figure 6A) nor in the color contrast and retention time (Figure 6B). These results were expected since no interaction between the electrochromic PEDOT:PSS layer and the substrate is present.

### 3.4 Application

**Figure 7:**
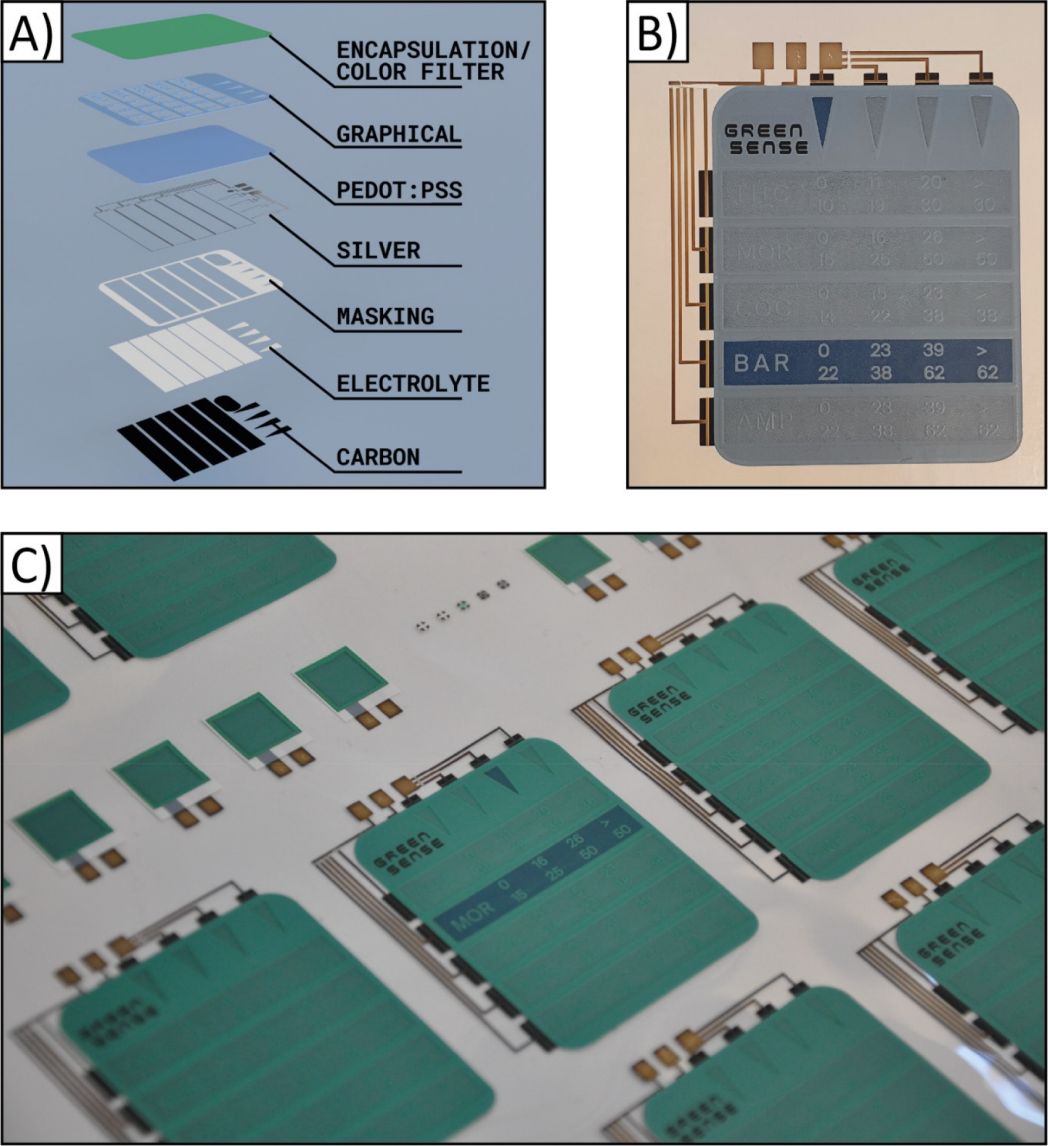
rOECDs used in the final application of the GREENSENSE project; a paper-based biosensor platform targeting drug of abuse detection. A) A more complex display design was required to indicate the different drug concentration levels. Each display consisted of a total of 9 segments, whereof 4 small arrows and 5 large rectangular segments, the latter also including text and numbers according to the design of the graphical layer. B) A screen printed rOECD on paper, without the color filter, in which the concentration of one of the drugs is indicated by two segments switched to their ON state. The switchable area of the rectangular segment is 44 × 7 mm^2^, while the base and height of the arrow segment is 3.5 and 8.5 mm^2^, respectively. The rOECD is direct addressed, which implies that 10 I/Os are needed in a microcontroller setup; one for each segment and an additional I/O for the common electrode. C) A sheet with multiple screen printed rOECDs including also the green color filter.

Within the GREENSENSE project (Horizon 2020 funded by EU),^47^ a paper-based biosensor platform for the detection of drug of abuse was envisaged. As part of the platform a screen printed OECD was planned as a visual indicator of the analytical response (drug concentration in the sample) of the PoC device. Since the whole sensor platform was printed on an opaque paper substrate, a reverse OECD architecture was required (Figure 7). To be able to display the different concentration levels of 5 sequentially measured drugs, a more complex rOECD design using 9 individually addressed segments was screen printed (Figure 7B); the 5 rectangular segments represent the drugs being monitored (tetrahydrocannabinol, morphine, cocaine, secobarbital, amphetamine) and the 4 arrows enable semiquantitative presentation of the drug concentration (ng mL^–1^). In this prototype a graphical lacquer was screen printed, in addition to the layers shown in Figure 1D, on top of the electrochromic PEDOT:PSS layer to create readable text and numbers in the segments. Printing of a graphical lacquer layer is an alternative avenue to patterning the masking layer, the latter is a more common method that has been performed in previous all-printed electrochromic displays. Additionally, to match the color scheme adopted in the GREENSENSE project, a green semitransparent lacquer can be screen printed as the last layer, as shown in Figure 7C.

## 4 Conclusions

In this report, we have presented all-printed reverse OECD architectures (rOECD) that open the possibility of using electrochromic technology for OECD applications on nontransparent flexible substrates. By screen printing the functional layers in reverse order (with the electrochromic PEDOT:PSS layer printed last) compared to conventional OECDs, rOECDs printed on paper substrates could be obtained with a manufacturing segment yield exceeding 99%. Upon applying an input voltage of 3 V, a display with an active area of 1 cm^2^ switches within 3 s. The new display architecture exhibits good color contrast (up to ΔE* ∼ 27), and up to 66% of the color contrast is retained even after 10 min in open-circuit mode. These performances were independent of the substrate used for the printing, as clearly elucidated by the comparison of rOECDs manufactured onto both paper and PET substrates. The rOECD experienced almost four times longer switching time compared to the conventional OECD and can only reach about 85% of the color contrast of the latter. However, the reverse architecture allows for electrochromic displays to be printed on paper, or other opaque substrates, and it has the additional advantage to have a faster absorption of moisture (approximately three times faster as compared to conventional OECDs). This is especially interesting in applications requiring storage in a very dry environment, for example, PoC devices, but that also need the OECD to be fully functional shortly after removal from their packaging. With the herein presented technology it is possible to produce relatively complex OECDs on paper substrates with several individually addressed segments displaying different messages. Additionally, since screen printing is used for the deposition of all layers, it is possible to scale up the manufacturing to larger volumes.

## Supporting Information Available

Dimensional instability of the paper substrate, design of screen printing tools, cycling stability of reverse organic electrochromic displays with respect to color contrast, and current vs time switching behavior (PDF)
