# Supplementary material for: Screen Printed Reflective Electrochromic Displays for Paper and Other Opaque Substrates
Source: ACS Appl Opt Mater. 2023 Jan 26;1(2):578–86. doi: 10.1021/acsaom.2c00140 (PMC9973558; doi:10.1021/acsaom.2c00140)
Supplement: Supplementary file 1 — ot2c00140_si_001.pdf [file ot2c00140_si_001.pdf]

Supporting Information

Screen printed reflective electrochromic displays for paper and other opaque substrates

Kathrin Freitag[1], Robert Brooke[1], Marie Nilsson[1], Jessica Åhlin[1], Valerio Beni[1], Peter Andersson Ersman[1]*

[1]RISE Research Institutes of Sweden, Digital Systems, Smart Hardware, Printed, Bio- and Organic Electronics, Bredgatan 33, SE-60221, Norrköping, Sweden

* E-mail: peter.andersson.ersman@ri.se

Paper expansion

The dimensional instability of the paper board used herein was investigated by its expansion following heat treatment. For this, two paper sheets were precured at 150 °C and the expansion of the paper board, when stored at room conditions (~20 °C / 50 %RH), was estimated by the misalignment of specific patterns obtained with UV curable inks at different time intervals (45, 90, 300 and 1260 minutes). As it can be seen from Figure S1 the paper board significantly expands when left at room conditions, even after 45 minutes (black curves). Importantly this effect could be minimized and somehow controlled (red lines) if a pre-heating treatment of 120 °C was introduced before every printing step.

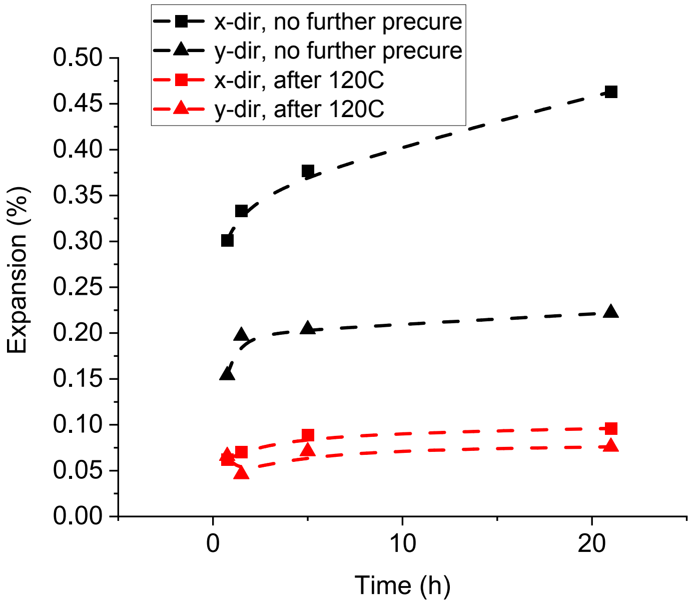

Figure S1. The expansion of the paper substrate in both planar directions as a function of time after the heat treatment at 150 °C. The black data shows the expansion without further treatment of the sheet. The red data shows the expansion when the sheets are precured at 120 °C before determining the expansion.

Design of the screen printing tools

The design layout used for screen printing of the rOECDs on paper substrates is illustrated in Figure S2.

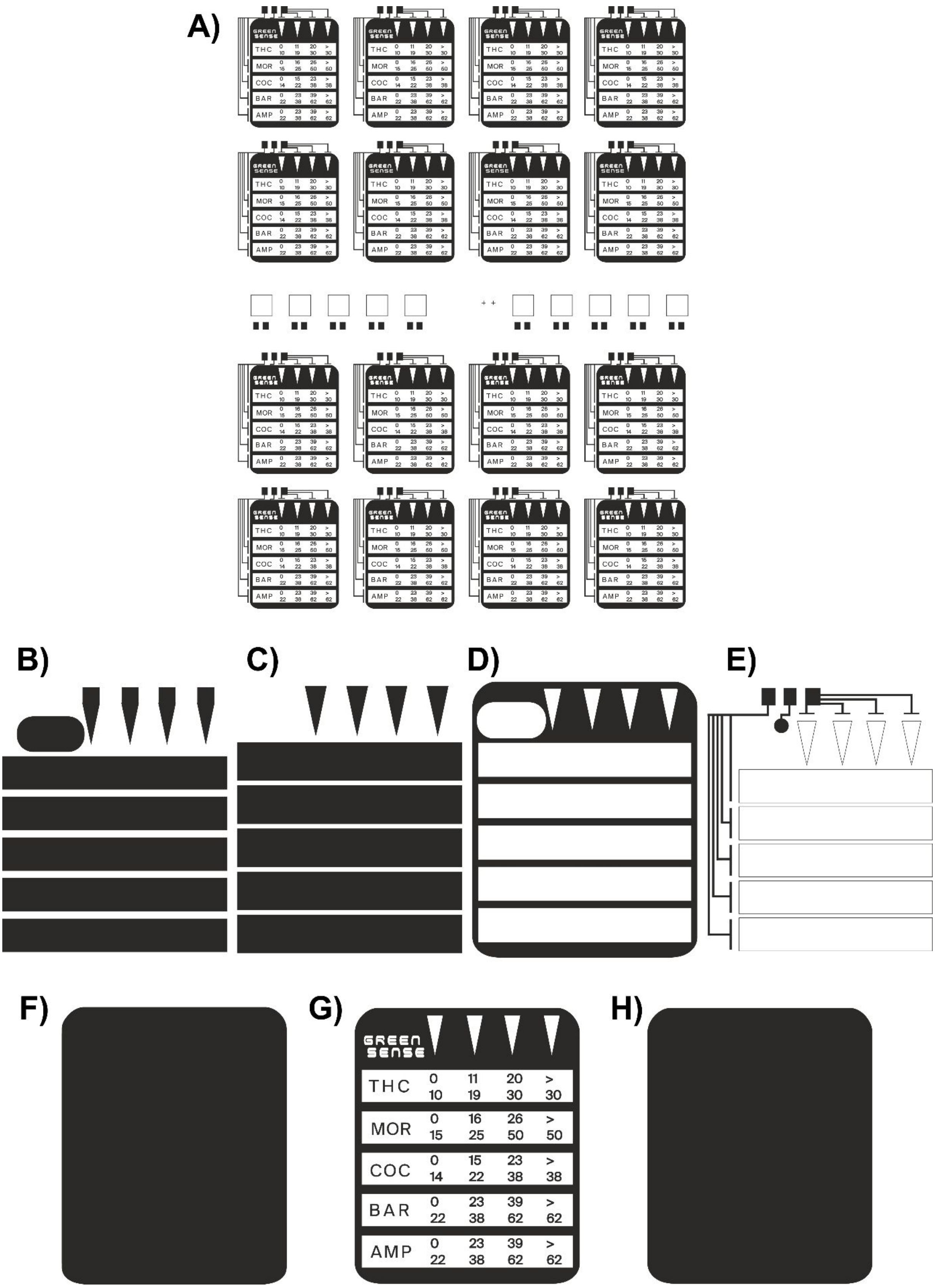

Figure S2. The design layout used for the screen printing of the rOECDs. A) The design layout of the whole screen, here illustrated by the silver and graphical layers, including two different display designs: a square shaped display with an active area of 10×10 mm for measurement purposes and a larger and more complex display design with 9 individual segments, *i.e.*, a total of 26 rOECDs are screen printed

on each sheet. The screen printed layers of the rOECD architecture are illustrated in the following schematics: B) the carbon counter electrode layer; C) the electrolyte layer; D) the masking layer; E) the silver layer; F) the electrochromic PEDOT:PSS layer; G) the graphic layer; H) the protection layer.

Cycling of the rOECDs

Both Type A and Type B rOECDs were switched ON and OFF multiple times. The initial color contrast was compared with that obtained after 100 switch cycles, see Table S1. Additionally, the initial electrical switching behavior (current vs. time) was compared with that obtained after 100 switch cycles, see Figure S3. The performances of the rOECDs, with respect to both color contrast and current vs. time switching behavior, did not change significantly upon cycling the display devices.

Table S1. 8 rOECDs were evaluated in this test, 4 of Type A and 4 of Type B. The color contrast (ΔE*) was measured initially and after 100 switch cycles. The table provides the relative change for each rOECD, expressed in percent (%). Only minor differences in color contrast are observed after cycling, and the positive values indicate a higher color contrast after 100 switch cycles. Hence, it is concluded that the color contrast is unaffected by the cycling.

Sample	Sheet #2, rOECD #1, Type A	Sheet #2, rOECD #2, Type A	Sheet #2, rOECD #3, Type A	Sheet #2, rOECD #4, Type A	Sheet #3, rOECD #1, Type B	Sheet #3, rOECD #2, Type B	Sheet #3, rOECD #3, Type B	Sheet #3, rOECD #4, Type B
Relative change in color contrast (% of ΔE*)	2.8 %	2.8 %	4.8 %	-1.2 %	-3.3 %	-0.4 %	1.2 %	0.7 %

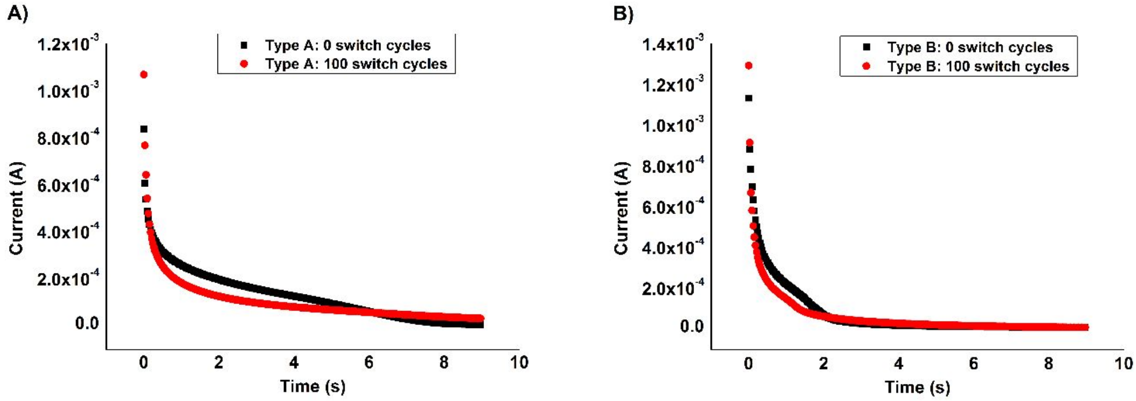

Figure S3. The current vs. time characteristics of the rOECDs when applying 3 V, both before (black) and after (red) 100 switch cycles for A) Type A and B) Type B rOECDs. Minor deviations can be observed in both device types when comparing the curves before and after 100 switch cycles, but neither these nor the differences in color contrast shown in Table S1 can be detected by the human eye.
